# Knowledge, attitudes, practices and willingness to vaccinate in preparation for the introduction of HPV vaccines in Bamako, Mali

**DOI:** 10.1371/journal.pone.0171631

**Published:** 2017-02-13

**Authors:** Anne S. De Groot, Karamoko Tounkara, Mali Rochas, Sarah Beseme, Shahla Yekta, Fanta Siby Diallo, J. Kathleen Tracy, Ibrahima Teguete, Ousmane A. Koita

**Affiliations:** 1 GAIA Vaccine Foundation, Providence, RI, United States of America; 2 Institute for Immunology and Informatics (iCubed), University of Rhode Island, Providence, RI, United States of America; 3 Foundation GAIA, Bamako, Mali; 4 Regional Director of Health, Bamako, Mali; 5 Departments of Epidemiology and Public Health, University of Maryland School of Medicine, Baltimore, Maryland, United States of America; 6 Hôpital Gabriel Touré, Bamako, Mali; 7 Laboratory of Applied Molecular Biology (LBMA), Science and Technologies Faculty (FST), University of Science Techniques and Technologies of Bamako (USTTB), Bamako, Mali; University of New South Wales, AUSTRALIA

## Abstract

Although screening for pre-cancerous cervical lesions and human papilloma virus (HPV) vaccination are accepted and effective means to prevent cervical cancer, women in Mali have limited access to these interventions. In addition, cervical cancer prevention by HPV vaccination has been controversial in some settings. To reduce cervical cancer prevalence and increase HPV vaccine uptake, it is important to understand the level of knowledge about cervical cancer screening and practices related to vaccination in at-risk populations. In this study, the level of knowledge about HPV and cervical cancer and attitudes towards vaccination were assessed among 301 participants (male and female, adults and adolescents) in a house-to-house survey in two urban neighborhoods in Bamako, Mali. The survey was combined with a brief educational session on HPV. Prior to the education session, overall knowledge of HPV infection and cervical cancer was very low: only 8% knew that HPV is a sexually transmitted infection (STI). Less than 20% of women had ever consulted a gynecologist and less than 3% had ever had cervical cancer screening. After hearing a description of HPV vaccine, more than 80% would accept HPV vaccination; fathers and husbands were identified as primary decisions makers and local clinics or the home as preferred sites for vaccination. This study provides information on STI knowledge and vaccine acceptance in Bamako, Mali in 2012, prior to the introduction of HPV vaccination.

## Introduction

Nearly 85% of worldwide cervical cancer (CC) cases diagnosed each year occur in low-resource countries. The main risk factor for CC is infection with high-risk types of human papillomavirus (HPV) [1]. In high-resource countries, prevention programs such as vaccination against HPV or CC screening have decreased the rates of CC and improved patient outcomes [2]. In contrast, the lack of access to early prevention measures such as gynecologic examination and cytology-screening programs, combined with a high incidence of HPV types 16 and 18 infection [3,4], contribute to the high prevalence of CC in some developing countries [5].

In West Africa, CC is the second most common and lethal cancer among women. A total of 27,300 new cases are identified in West Africa every year, and 16,500 deaths per year are attributed to CC [6]. High-risk HPV types 16 and 18 are found in 60% to 70% of invasive cervical cancer (ICC) cases in Africa [7]. Mali, a West African country with an estimated population of 15 million (4.08 million women aged 15 and over), has the highest regional rate of CC, with an age-standardized rate of 44.2 cases per 100,000 women [8,9]. The prevalence of high-risk HPV types has been estimated at 12% among previously unscreened women aged 15–65 [10,11]. Previous studies conducted in Mali showed that high-risk HPV types 16 and 18 could be identified in 54% to 62% of CC cases [12,13]. Unfortunately, according to household surveys performed by the WHO in 2000–2001, cervical cancer screening coverage is estimated at 4.8% (women aged 18–69 years) [8].

As of 2015, HPV vaccines are only available through pilot programs in some regions of Bamako prior to countrywide HPV vaccine introduction. At the time this study was performed (2011–2013), HPV vaccine was not available, but plans were underway to bring the HPV vaccine to Mali. The GAVI alliance recently initiated a pilot program (2014–2016). This program targeted 12,500 10-years old girls in Fana and Commune V of Bamako. The vaccination includes three injections administered at school or in health centers and through community outreach. Scale up is planned for late 2016 or early 2017. In anticipation of the introduction of HPV vaccination and the expansion of current CC screening programs in Mali, it is important to assess the willingness of Malians to be vaccinated and the level of knowledge about the link between HPV and CC. Recent studies have shown that one of the barriers to the delivery of CC screening is the lack of awareness about the disease [14]. Education campaigns have been shown to increase the level of acceptance of vaccines such as the polio vaccine [15]. Furthermore, HPV vaccination is currently recommended for adolescent girls aged 9–12 [16], for whom autonomous decision-making is limited. Acceptance of vaccination is dependent on many factors, such as individual knowledge, beliefs about susceptibility, perceptions of vaccine effectiveness, family and physician perspectives, sexual and cultural practices, and anticipated costs of vaccination [17]. We therefore assessed the knowledge, attitudes, and practices regarding CC and HPV among teenagers and adults living in two urban regions of Bamako, Mèkin-Sikoro and Djikoroni.

## Methods

### Recruitment of participants

Participants were recruited between May and June 2011 in Mèkin-Sikoro and Djikoroni, two peri-urban communities of Bamako that have been site for previous population surveys by our group [17]. Briefly, a team of four trained interviewers (one adolescent female, one adolescent male, one adult female, and one adult male) approached members of households separately. In Mékin-Sikoro, the selection of households proceeded within the six districts in the neighborhood as follows: the first interviewer approached the closest household to the central health clinic, the second interviewer approached the next closest household, and so on. In Djikoroni, households were selected in advance based on the age of the residents. According to the study protocol, if at least one eligible participant matching the sex and age group of the interviewer was present at the time of visit, the household was entered into the study. Adolescents were eligible if aged 12–17 years and living in the household, and if a guardian was available to provide consent. Adults were eligible if aged ≥18 years and living in the household. Participation in the study was explained and proposed to the first eligible individual to interact with the age and sex- matching interviewer within a household. A total of 301 participants (150 living in households in Djikoroni, 151 living in Mékin-Sikoro) agreed to participate in this study and signed (or their guardian signed) an informed consent form.

### Assessment of knowledge, attitudes and practices towards HPV and CC and willingness to participate in an HPV vaccine trial

Two standardized questionnaires were used in the structured interviews. These questionnaires were pilot-tested in March 2011 [17], and were adapted for relevancy to each sample subset. Interviews were conducted in participants’ home. When a household was approached, the interviewer engaged discussion with an age and gender- matched member of the household, i.e. a younger (18–25 year old) female interviewer would interact with the adolescent female in the household; older female interviewers were matched with older women, etc. Questions were asked by the interviewers, who filled out the questionnaires. A guardian was present when adolescents were interviewed. After the study purpose was explained and informed consent obtained, the first questionnaire was submitted to the participants to assess knowledge, attitudes and practices; vaccine acceptability; willingness to participate in an HPV vaccination program; and vaccination decision-making capacity. The detailed questionnaire is available in supporting information file 1 (S1 File). Demographic characteristics and information about marital status and sexual experiences and behaviors were also collected. An information and education session took place after the first interview, consisting of information about HPV and CC, their symptoms and causes, and the availability of CC testing in Mali. After this educational session, a second questionnaire was administered to measure immediate information retention and comprehension from the education session. The detailed questionnaire is available in supporting information file 2 (S2 File). The structured interviews were conducted in Bambara, the main language in Mali, following a script that was provided to the interviewers in French.

### Statistical analysis

Individual results from the surveys was hand-entered into a spreadsheet and then collated into summary tables (Microsoft Excel). All statistical analyses were performed using GraphPad (http://www.graphpad.com). In order to evaluate the impact of the education session, we compared answers before and after the education session with a McNemar test that tests the likelihood of a change in binary response after an intervention to be random. p<0.01 was considered significant.

### Ethics statement

The protocol of this study and the surveys were reviewed and approved by the Committee of Ethics of the Faculty of Medicine, Pharmacy and Odonto-stomatology in Bamako, Mali, and by the Ethical and Independent Review Services Midwest Board, Independence, MO, United States. The approved informed consent document was translated from French to Bambara, the local language. The consent form was read aloud to individuals who were illiterate. All participants provided a signature or fingerprint indicating their informed consent. Written informed consent was obtained from the next of kin, caretakers, or guardians on behalf of the minors/children enrolled in the study. All interviewers were trained with information about HPV and Cervical cancer and ethics considerations. Informed consent forms and training manual are available in supporting information files 3 and 4 respectively (S3 and S4 Files).

## Results

### Characteristics of the study participants

The characteristics of the participants are summarized in Table 1. Of the total eligible participants approached, 301 (100%) elected to participate in the structured interview. Half of the households were located in Sikoro and the other half in Djikoroni, two densely inhabited and low-income neighborhoods of Bamako. The majority of participants had attended some school (n = 229; 76.1%). Male participants reported more sexual partners before marriage (average 7.8) than women (average 1.4). Of note, among those reporting having had sex 26 (of 176, or 14.7%) reported having been forced to have sex against their will (17.2% of females; 12.4% of males). Fourteen individuals (8.0%; 6 females, 8 males) reported having had sex in exchange for money or gifts.

**Table 1:** Characteristics of the study participants.

		Total (N = 301)		Female (N = 149)		Male (N = 152)	
		N	%	N	%	N	%
**Mean Age (SD)**		22.0 (9.7)		21.6 (9.5)		22.3 (9.9)	
	Age (years) 12–17	146	48.5	74	49.7	72	47.4
	18–26	84	27.9	39	26.1	45	29.6
	>26	71	23.6	36	24.2	35	230
**Circumcised/excised**^**‡**^		292	97.0	141	94.6	151	99.3
**Ever attended school**		229	76.1	103	69.1	126	82.9
**Married**		87	28.9	59	39.6	28	18.4
	Adolescents (<18 years)	6	4.1	6	8.1	0	0.0
	Adults (≥18 years)	81	52.3	53	70.7	28	35.0
	Polygamous marriage*	11	12.6	10	17.0	1	3.6
	Average number of wives^†^ (SD)	2.0 (0.6)		2.0 (0.7)		2.0 (N/A)	
**Have had sexual intercourse**		176	58.5	87	58.4	89	58.6
	Mean age of sexual debut^§^ (SD)	17.4 (4.4)		16.5 (2.5)		18.4 (5.4)	
	Age of sexual debut^§^ (years) <15	30	17.0	17	19.5	13	14.6
	15–18	82	46.6	47	54.0	39	39.3
	>18	64	36.4	23	26.4	41	46.1
	Mean number of sexual partners^§^ (SD)	3.0 (5.3)		1.6 (1.2)		4.3 (7.2)	
	Mean number of sexual partners before marriage^#^ (SD)	4.0 (8.8)		1.4 (0.8)		7.8 (13.1)	
	Forced to have sex^§^	26	14.7	15	17.2	11	12.4
	Sex in exchange for money or gifts^§^	14	8.0	6	6.9	8	9.0
**Know what contraception is**		—		87	58.4	—	
Use some form of contraception^**^		—		37	42.5	—	
	Condoms ^††^	—		3	8.1	—	
	Birth control pill ^††^	—		15	40.5	—	
	Birth control injection ^††^	—		9	24.3	—	
	Other ^††^	—		11	29.7	—	

Note: SD = standard deviation, N/A = Non-Applicable

‡ Excision refers to Type II female genital cutting, or the removal of the clitoris and inner labia.

* Among those who were married; N = 87 for total; N = 59 for female; N = 28 for male.

† Among those who were in a polygamous marriage; N = 11 for total; N = 10 for female; N = 1 for male.

§ Among those reporting having had sex; N = 176 for total; N = 87 for female; N = 89 for male.

# Among those who were married, reporting having had sex before marriage; N = 42 for total; N = 25 for female; N = 17 for male.

** Among those who knew what contraception is; N = 87.

†† Among those who use contraception; N = 37.

### Knowledge of STIs, HPV, and CC

**Fig 1:**
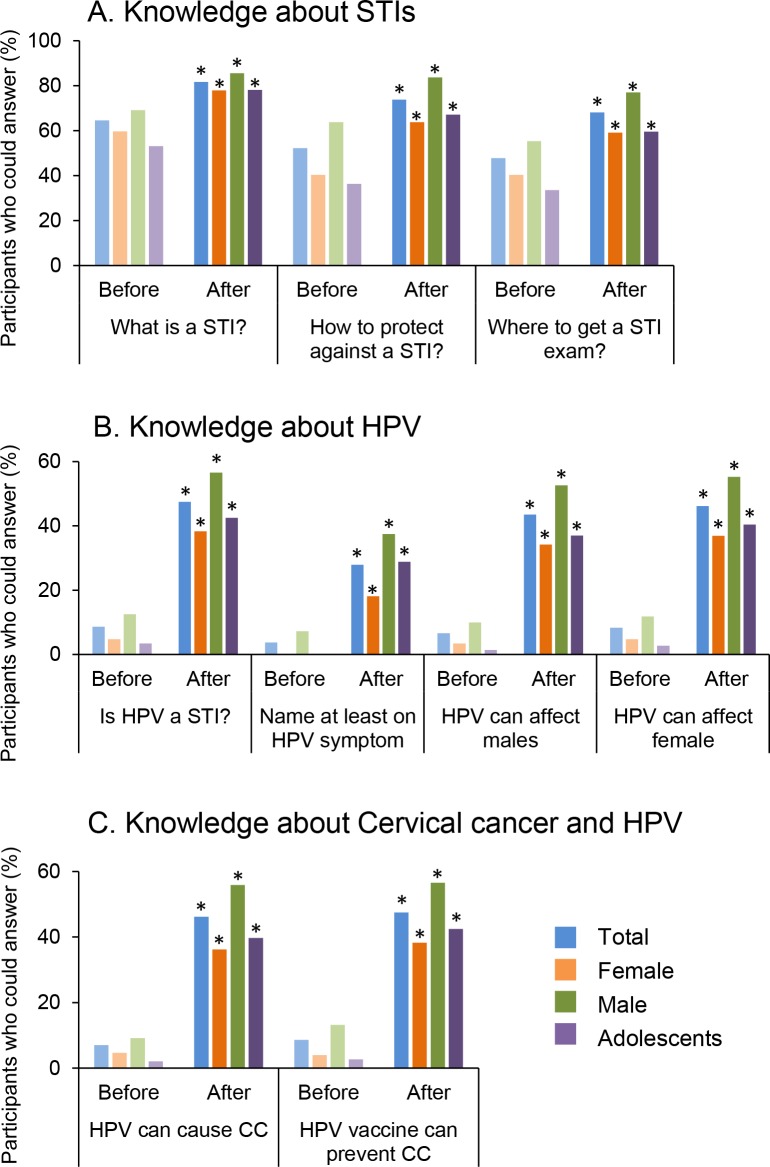
Knowledge of STIs, HPV, and CC. Participants were asked about STIs (A), HPV (B) and cervical cancer (C) before and after a brief educational session. Percentages of participants able to answer the question are reported in the graphs for all participants (Total, N = 301), female (N = 148), male (N = 152) and adolescents (N = 146). * p<0.01 when compared to answers before the educational session (McNemar test). Women reported much more extensive knowledge of STIs than HPV, and while a brief educational session improved knowledge, more needs to be done.

Before the educational session, 194 participants (64.5%) reported they knew what a sexually transmitted infection (STI) was (Fig 1A). Forty-six participants (15.3%) reported having a STI in the past (data not shown), while 157 participants (52.2%) could correctly identify a method of protection against STIs and only 144 (47.8%) could correctly identify a place where STI exams were offered. Awareness about HPV was much lower: only 26 participants (8.6%) knew that HPV was transmitted by sexual contact (Fig 1B). None of the female subjects were able to correctly describe at least one symptom related to HPV. Only 21 participants (7%) correctly stated that HPV was one of the major causes of CC (7 females, 14 males) prior to the educational session (Fig 1C). Overall, knowledge about STIs, HPV, and CC was better among males than females. Adolescents demonstrated very poor knowledge: only 5 (3.4%) knew that HPV is a STI.

The knowledge of the participants significantly increased after the education session. Notably, all participants who knew that HPV was a STI correctly said that HPV vaccination could prevent CC (Fig 1C). On average, the number of participants who could correctly answer the questions asked increased by 30% after the brief education session.

### Access to gynecological care

**Fig 2:**
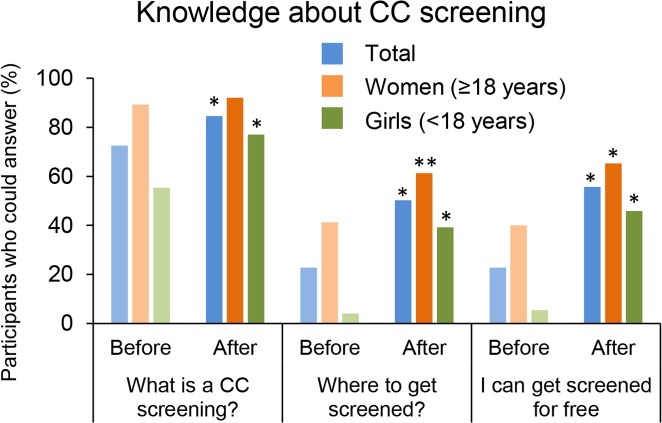
Knowledge about CC screening. Female participants (N = 149) were asked if they knew what CC screening was and about access to CC screening in Mali. Results are presented before and after the educational session for all female participants (Total, N = 149), female adults (Women, N = 75) and female adolescents (Girls, N = 74). * p<0.01 and ** p<0.05 when compare to answers before the educational session (McNemar test).

Questions related to gynecological information were asked to female participants. Only 31 (20.8%) consulted with a provider for gynecological issues at least once (Table 2). Although cervical cancer screening using visual inspection with acetic acid and Lugol’s iodine (VIA and VILI) is available in Mali, very few women and adolescent girls knew what this test was (N = 10, 6.7%), and only one-third (2.0% overall) reported that they had ever received such an exam (Table 2). Only 41 women and 21 girls (54.7% and 28.4%, respectively) had heard of CC, and 6 participants could name at least one CC symptom. Adult women were well aware of what was CC screening but only 34 (22.8%) knew where to get screened before the educational session (Fig 2).

**Table 2:** Gynecological care knowledge and practices (female participants).

	Total (N = 149)	Women*	Girls^†^ (N = 74)
	N (%)	(N = 75) N (%)	N (%)
Has been evaluated for gynecology problems	31 (20.8)	26 (34.7)	5 (6.8)
Knows what a CC screening is (VIA/VILI)	10 (6.7)	5 (6.7)	5 (6.8)
Has ever had a CC screening (VIA/VILI)	3 (2.0)	2 (2.7)	1 (1.4)
Has heard of CC	62 (41.6)	41 (54.7)	21 (28.4)
Knows at least one CC symptom	6 (4.0)	4 (5.3)	2 (2.7)
Knows that CC can cause death in women	93 (62.4)	68 (90.6)	25 (33.8)

* Includes female adults aged 18 years or older.

† Includes female adolescents aged 12–17 years.

### Willingness to vaccinate against HPV and vaccination preferences

**Fig 3:**
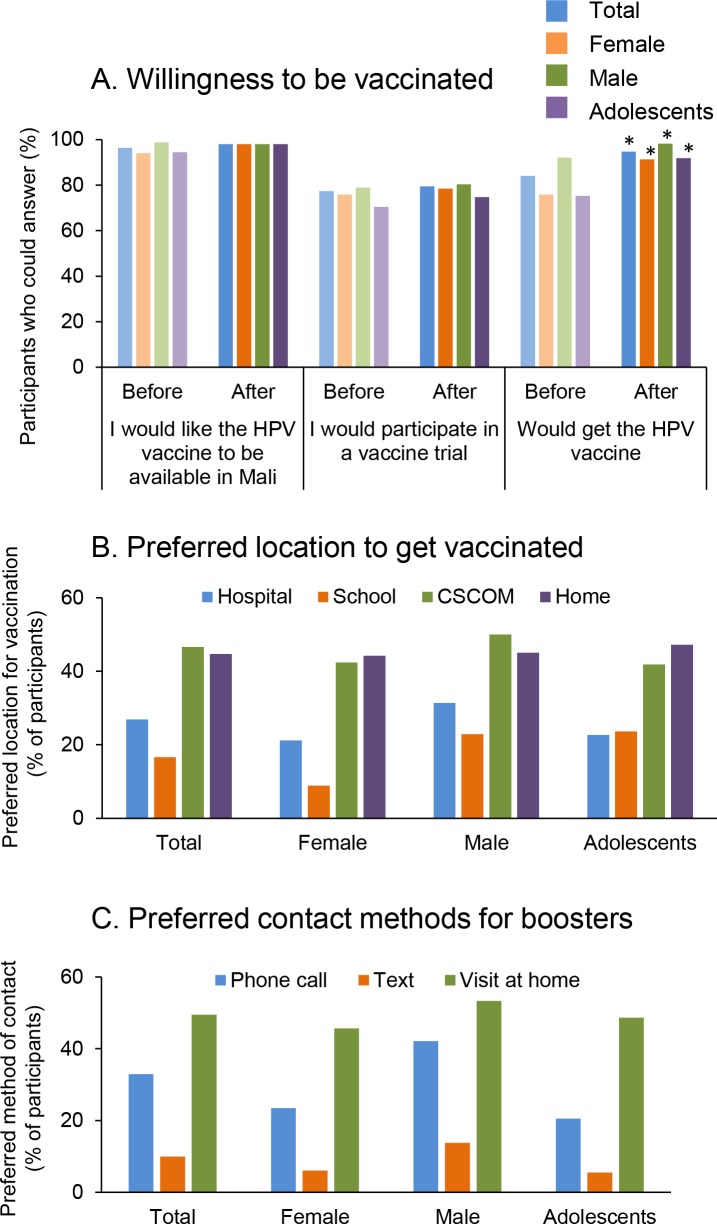
Willingness to be vaccinated against HPV and immunization preferences. A. Willingness to participate in a vaccine trial and to get vaccinated was assessed before and after the educational session for all participants (Total, N = 301), female (N = 149), male (N = 152) and adolescents (N = 146). * p<0.01 and ** p<0.05 when compare to answers before the educational session (McNemar test). B, C: The preferred location to get the vaccine and the preferred method of contact for booster was assessed among participants who would choose to get vaccinated (Total, N = 253; Female, N = 113; Male, N = 140, Adolescents, N = 110). Interest in vaccination was high, two locations were preferred (local clinics (CsCOMS) and at home), and home visits rather than phone messages were preferred for reminders.

Vaccine acceptability was high among the participants. Nearly all participants had received a vaccine before (N = 296; 98.3%, data not shown). A majority of participants (N = 290, 96.3%) said they would like the HPV vaccine to be available in Mali and 233 (77.4%) said that they would participate in a vaccine trial (Fig 3A). The education session increased the HPV vaccine acceptance in all groups, especially among adolescents (from 75.3% to 91.8%, Fig 3A). Among all groups, the preferred locations to receive the vaccine were community clinics (CSCOM) and the participants’ homes (Fig 3B). Home visits (N = 149; 58.9%) were preferred over SMS or phone calls (N = 99; 39.1%) for the purposes of receiving information about HPV vaccination appointments (Fig 3C).

### Ability to autonomously decide to vaccinate

**Fig 4:**
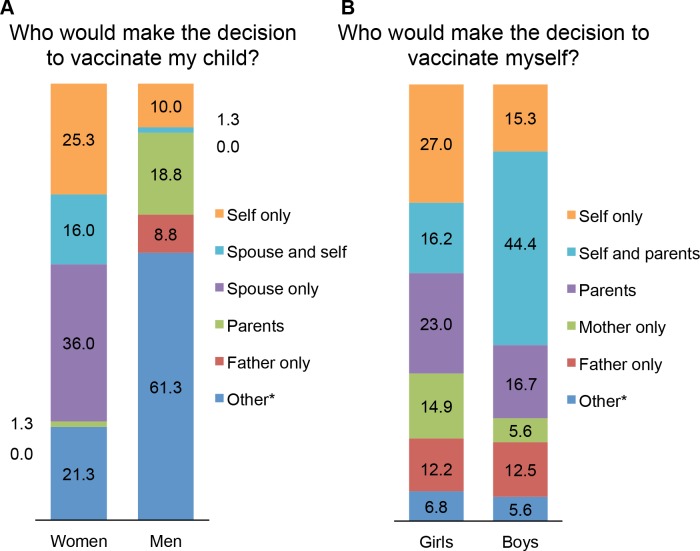
HPV Vaccination Decision-making Autonomy. Participants were asked who would participate in the decision of vaccinate their children (adults, A) or themselves (adolescents, B). Results are shown as percentage of female ≥ 18 years (Women, N = 75), male ≥ 18 years (Men, N = 80), female <18 years (Girls, N = 74) and male <18 years (Boys, N = 72). * “Other” includes participants who did not answer the question. Women reported less autonomy regarding vaccine decision-making than men regarding vaccination for themselves and their children.

The ability to autonomously decide to vaccinate oneself or their child with an HPV vaccine was significantly different across gender and age groups (Fig 4). Half of adult females (39, 52.0%) said they would have to ask their husband’s permission before deciding to vaccinate their child. However, most adult men (88.9%) were not able to decide or would ask their parent’s opinion. Only one adult male participant (1.3%) reported that he would consult his wife about HPV vaccination. Among adolescents, boys would be more inclined to ask permission from their parents, but overall they would be more involved in the decision to get vaccinated (59.7%) than girls (43.2%).

## Discussion

This study assessed knowledge towards HPV, CC, and HPV vaccination in a representative sample of the community in two peri-urban neighborhoods of Bamako, Mali before the implementation of HPV vaccination and widespread availability of CC screening in the country. We also assessed vaccination decision autonomy and preferred vaccination locations.

In a parallel study, our group recently demonstrated that the two high-risk HPV 16 and 18 types were found in most of the CC biopsies in a group of patients from a main hospital in Bamako (manuscript submitted contemporaneously). Modeling studies have shown that vaccination against HPV 16 and 18 by either of the currently available bi or quadri-valent HPV vaccines could reduce the prevalence of HPV-associated CC in Mali by a rate directly proportional to the coverage of the population. For example, 15% vaccination coverage would reduce the maximum prevalence of infection from 39% to 33%, whereas 90% coverage would reduce the peak prevalence of infection to 7% [18]. Based on available information on the prevalence of HPV in Mali and the cost of vaccination, the introduction of HPV vaccine in Mali would be cost-effective [11].

Among all subsets of the population engaged in this survey, knowledge of vaccination and willingness to vaccinate themselves and/or their children against HPV was high. Most of the participants would be willing to participate in a HPV vaccine trial. This was consistent with a prior study conducted by our group, in which 279 individuals who were approached in market places in Bamako Mali were surveyed about vaccine trial participation: 75% were willing to participate to a vaccine trial for tuberculosis, 70% for malaria, and 62% for human immunodeficiency virus (HIV) [unpublished results].

Most of the participants reported that they were 15–18 years of age at sexual debut, indicating that the current targets for HPV vaccination (girls aged 9–12 years [16]) would be appropriate in this setting. Indeed, since first sexual encounters seem to occur somewhat later relative to developed countries, it may be possible to extend the target vaccination age higher in Mali (to 15 years of age) than the range used elsewhere.

Sexual coercion and sexual violence, which put women at risk of HPV infection and other STIs, appear to be common in this population. Overall, 14.7% of sexually active participants reported having had forced sex (8.6% of all participants) at least once, including both females and males. Self-reporting (in the participants’ home) may underestimate the true prevalence. Studies in South Africa reported similar results, and surveys among young girls’ mothers revealed that the risk of forced sex or sexual violence is a factor that positively influences their willingness to vaccinate their child against HPV infection [19,20].

Our findings demonstrate that potential vaccination participants prefer to be contacted and to receive vaccinations by home visits or at their local CSCOM (community clinic). This is consistent with current practices in Mali, as vaccination campaigns are conducted at the community level, either in the CSCOM or in house-to-house campaigns for polio and measles [21]. In these settings, parental permission to vaccinate girls against HPV could be obtained during the clinic or home visits; in addition, methods to monitor vaccination completion at the individual and neighborhood level, such as registries and databases, could be used [22].

Although high HPV vaccine coverage rates can be achieved through school-based delivery strategies in adolescent girls (age 9–14) in low and middle-income countries [23], this strategy poses a challenge for reaching those who do not attend school. Door-to-door vaccination campaigns have been used as an effective strategy for delivering vaccines to hard-to-reach populations [24]. Prospective studies could be performed to compare clinic (CSCOM)-based, household-based, and school-based vaccination in terms of coverage rates for the two to three dose HPV vaccination that is currently recommended [16].

In this study, home visits were the preferred method of contact for participants. Phone calls were preferred over text messages by all participants suggesting that contacting study participants through text reminders for second and third doses in a three-dose regimen may not transfer to Mali at this time. This may be due to low literacy rates: only 31% of Malians over age 15 are literate (2010 data from the WHO), meaning that 69% of the target population would be unable to read a text message on a phone.

Another factor to be considered when designing a prevention program will be the autonomy to make a health-related decision. This study is, to the best of our knowledge, is the first large-scale study to examine the differences in permission required to receive HPV vaccination in Mali. Female participants identified men (husbands and fathers) as the primary decision-makers regarding HPV vaccination. Men had significantly greater autonomy in the decision to vaccinate themselves than women and adolescents (p = 0.005). These findings were somewhat expected, as Mali has a patriarchal decision-making structure [25]. Our results suggest that men’s opinions will be an important factor to consider when implementing vaccination campaigns.

Although 52% of women need the approval of their husband to vaccinate their child, most men would ask their parents or could not answer. Engagement of husbands has been shown to be a critical factor for the delivery of CC screening in Malawi [14]. Therefore, involving men in education about prevention programs, engaging men in making positive health decisions for women and children, and including men in publicity materials will be important to promote prevention programs not only in Mali, but also in other societies where men are heavily involved in decisions about women’s health.

Critical issues that remain to be addressed to improve the situation regarding CC prevalence and limited access to CC screening in Mali and West Africa include the following: (1) low levels of HPV and CC knowledge; (2) limited knowledge about the purpose and location of cervical cancer screening, and (3) lack of access to cervical cancer screening. We are currently evaluating methods for increasing participation in HPV/CC screening using community education sessions and a patterned cloth as a visual teaching tool.

It is clear from this study that HPV vaccination campaigns should be carefully structured, taking into consideration local cultural norms. Autonomy regarding vaccination was low among women and girls, thus, future studies should examine whether HPV vaccination campaigns should be conducted in clinical settings or at home, and how decisions about HPV vaccination, that are made in consultation with fathers or husbands, could be facilitated.

In conclusion, this study provides information to guide future interventions such as HPV vaccination, CC screening campaigns, and publicity campaigns designed to improve HPV knowledge and vaccine acceptability in West Africa. Our results may be useful to support the implementation of vaccines against HPV in Mali. Implementation of HPV vaccination in Mali (and West Africa) would avert tens of thousands of future deaths. Perhaps most important, HPV vaccination is desired and would be accepted by the vast majority of participants in this study.

## Supporting information

S1 File Questionnaire before education session (English). Questions asked before the education session are listed in S1 file. Every participant was asked questions 1–10, 21–31 and 41–50. Only female participants were asked questions 11–20 and 32–40. These questions are in bold within the questionnaire. Questions highlighted in green were asked again during the second interview after the education session.
(DOC)

S2 File Questionnaire after education session (English). Questions asked after the education session are listed in S2 file. Every participant was asked questions 1–10 and 18–30. Only female participants were asked questions 11–17. Questions highlighted in green were also asked during the first interview before the education session.
(DOC)

S3 File Informed Consent (French). Age-adapted informed consent forms were used in this study. Page 1–2 of S3 file shows the consent form used for adults > 18 years old. Pages 3–6 shows consent forms proposed to adolescents < 18 years old and their legal guardian.
(DOC)

S4 File Training Manual for staff members (French). A three- days training was provided for interviewers before initiation of the study. The training manual is displayed in S4 file, in French.
(DOCX)
