# Supplementary material for: Knowledge, attitudes, practices and willingness to vaccinate in preparation for the introduction of HPV vaccines in Bamako, Mali
Source: PLoS One. 2017 Feb 13;12(2):e0171631. doi: 10.1371/journal.pone.0171631 (PMC5305061; doi:10.1371/journal.pone.0171631)
Supplement: S1 File — Questions asked before the education session are listed in S1 file. Every participant was asked questions 1–10, 21–31 and 41–50. Only female participants were asked questions 11–20 and 32–40. These questions are in bold within the questionnaire. Questions highlighted in green were asked again during the second interview after the education session. (DOC) [file pone.0171631.s001.doc]

##
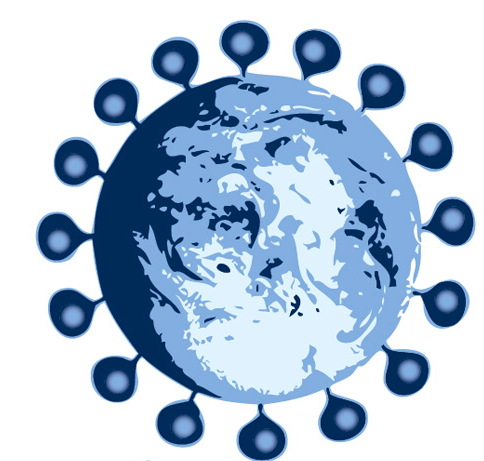
GAIA

**Global Alliance to Immunize against AIDS**

**146 Clifford Street (401) 453-2068**

**Providence, RI 02903 USA** [**www.GAIAvaccine.org**](http://www.GAIAvaccine.org/)

**Knowledge, Attitudes and Practices Study related to HPV and cervical cancer and Willingness to Participate in an HPV vaccine trial in the region of Bamako, Mali, West Africa**

Date :

ID Code :

**FIRST QUESTIONNAIRE**

**Part I : Background information about the participant**

1. How old are you?:

______years

 Prefer not to answer

1. Marital status: Are you:

 Married

 Married in a polygamous family If yes: How many wives are there?

 Cohabiting

 Single

 Prefer not to answer

1. (a) Did you go to school? :

 Yes

 No (Go directly to Part II)

3. (b) If yes, until what age? :

3. (c) or what grade?:

**Part II: Sexual history**

1. Are you circumcised?

 Yes

 No

 Prefer not to answer

 Don’t know

1. (a) Have you ever had a sexual relationship?

 Yes

 No *(For men and adolescent boys go directly to Part IV)*

*(For women and adolescent girls go directly to Part III)*

 Prefer not to answer

 Don’t know

5. (b) How old were you when you had your first sexual relationship? :

 Years old

 Prefer not to answer

 Don’t know

1. How many sexual partners have you had? :

 Partners

 Prefer not to answer

 Don’t know

1. Have you ever been forced to have sexual relations?

 Yes

 No

 Prefer not to answer

1. Have you had sexual relations in exchange for something?

 Yes for money

 Yes for food

 Yes for shelter

 Yes for other reasons,(please elaborate): _______________________

 No

 Prefer not to answer

If the person is married:

1. How many sexual partners did you have before getting married? :

 Partners

 Prefer not to answer

 Don’t know

1. Do you have – or did you have - any sexual partners other than your husband?

 Yes

 No

 Prefer not to answer

***FOR MEN AND TEENAGE BOYS GO DIRECTLY TO PART 4***

**Part III - Gynecological information**

***FOR WOMEN AND ADOLESCENT GIRLS ONLY***

1. **(a) Are you menstruated?**

 Yes

 No  *(Go directly to Q14)*

 Prefer not to answer

 Don’t know *(Go directly to Q12)*

**11. (b) How old were you when you began menstruating?**

 Prefer not to answer

 Don’t know

1. **(a) Do you have children? :**

 Yes

 No *(Go directly to Q13)*

 Prefer not to answer *(Go directly to Q13)*

 Don’t know *(Go directly to Q13)*

**12. (b) How many children do you have?**

 children

 Prefer not to answer

 Don’t know

1. **(a) How many pregnancies have you had?**

 pregnancies *(if none, Go directly to Q14)*

 Prefer not to answer

 Don’t know

**13. (b) How old were you when you had your first pregnancy?**

 years

 Prefer not to answer

 Don’t know

1. **(a) Have you ever gone to a gynecologist? for reasons other than pregnancy care?**

 Yes

 No *(Go directly to Q15)*

 Prefer not to answer *(Go directly to Q15)*

 Don’t know *(Go directly to Q15)*

**14. (b) How many times have you gone to a gynecologist?**

 Times

 Prefer not to answer

 Don’t know

**14. (c) For what reasons?**

 Pain

 Itching

 Abnormal bleeding

 Vaginal discharge

 Loss of menstrual periods

 Others: _____________________

 Prefer not to answer

 Don’t know

1. **Do you know what a Pap smear is?**

 Yes

 No

 Prefer not to answer

If no, explain what a Pap smear is: *A gynecological Pap smear is a procedure that uses an instrument (a spatula) to collect and examine cervical cells for health reasons.*

1. **(a) Have you ever gotten a Pap smear?**

 Yes

 No *(Go directly to Q17)*

 Prefer not to answer *(Go directly to Q17)*

 Don’t know *(Go directly to Q17)*

**16. (b) How old were you when you got your first Pap smear?**

 years

 Prefer not to answer

 Don’t know

**16. (c) If yes Where did you have the Pap smear done?**

 At the hospital

 At the CSCOM

 At a free testing center

 At home

 Prefer not to answer

 Don’t know

**16. (d) Who performed this Pap smear?**

 A gynecologist

 A midwife

 Other: Explain:

 Prefer not to answer

 Don’t know

1. **Do you know what contraception means?**

 Yes

 No *(Go directly to Q19)*

 Prefer not to answer *(Go directly to Q19)*

1. **Do you use a method of contraception?**

 Yes Which one(s)? :

 No

 Prefer not to answer

 Don’t know

1. **How frequently do you wash your genitals?**

 Never *(Go directly to Part IV)*

 Once a day

 Less than once a day

 More than once a day

 Prefer not to answer *(Go directly to Part IV)*

 Don’t know *(Go directly to Part IV)*

1. **(a) Do you use any feminine hygiene products?**

 Yes

 No

 Prefer not to answer

 Don’t know

**20. (b) If yes to question: Which product(s) do you use?**

 Soap

 Other (Explain: )

 Prefer not to answer

 Don’t know

**If no to question : What do you use?**

 Water

 Other (Explain: )

 Prefer not to answer

 Don’t know

**Part IV – STIs and HPV**

1. Do you know what an STI is?

 Yes

 No *(Go directly to Q26)*

 Prefer not to answer

1. Do you know how to protect yourself against STIs?

 Yes (Explain: )

 No

 Prefer not to answer

1. Do you know where to go to get tested for STIs?

 Yes (Where : )

 No

1. Have you ever had an STI?

 Yes: Which one (s)?:

 No *(Go directly to Q26)*

 Prefer not to answer *(Go directly to Q26)*

 Don’t know *(Go directly to Q26)*

1. How many times have you had an STI?:

 times

 Prefer not to answer

 Don’t know

1. Do you know what HPV is?

 Yes: Please elaborate:

 No *(Go directly to part V)*

 Prefer not to answer

*HPV is the human papilloma virus. It is an STI that is common among sexually active people.*

1. Do you know one or more of the symptoms of HPV?

 Yes : Which ones :

 No

1. Please chose among the following answers:

 HPV affects women?

 HPV affects men?

 HPV affects adolescent girls ?

 HPV affects adolescent boys?

 Prefer not to answer

 Don’t know

1. Is HPV related to cervical cancer?

 Yes

 No

 Prefer not to answer

 Don’t know

1. Is HPV one of the main causes of cervical cancer?

 Yes

 No

 Prefer not to answer

 Don’t know

1. (a) Can cervical cancer be avoided if one is vaccinated against HPV?

 Yes

 No

 Prefer not to answer

 Don’t know

(b) Among women, is HPV one of the main causes of cervical cancer?

 Yes

 No

 Prefer not to answer

 Don’t know

***FOR MEN AND ADOLESCENT BOYS GO TO PART VI***

**PART V – Cervical cancer**

***FOR WOMEN AND ADOLESCENT GIRLS ONLY***

1. **Have you heard of cervical cancer?**

 Yes

 No

 Prefer not to answer

 Don’t know

1. **Do you know one or more of the symptoms or signs of cervical cancer?**

 Yes: Which ones?

 No

 Prefer not to answer

 Don’t know

1. **Have you heard that there is free testing for cervical cancer in Mali?**

 Yes

 No

 Prefer not to answer

1. **Do you know where to go to get tested (i.e. get a cervical exam)?**

 Yes : Where ?;

 No

 Prefer not to answer

1. **Have you ever been tested (i.e. gotten a cervical exam)?**

 Yes: Where? How many times?

 No *(Go directly to Q37)*

 Prefer not to answer

1. **Do you know what happens during a cervical exam?**

 Yes

 No

 Prefer not to answer

1. **True/False: A screening test tells you if you have cervical cancer**?

 True

 False

 Don’t know

 Prefer not to answer

1. **True or False: Cervical cancer is a cause of death among women?**

 True

 False

 Don’t know

 Prefer not to answer

1. **Do you know one or more of the causes of cervical cancer?**

 Yes: Which ones?:

 No

 Don’t know

 Prefer not to answer

**Part VI – Vaccines and vaccine trials**

1. Have you ever been vaccinated?

 Yes: list the names of the vaccines you received:

 No

 Prefer not to answer

 Don’t know

1. If the HPV vaccine were available in Mali, to whom should it be given? (Mark your response for each of the following groups.)

(a) To young adolescent girls before their first sexual relationships?

 Yes

 No

 Prefer not to answer

 Don’t know

(b) To young adolescent boys before their first sexual relationships?

 Yes

 No

 Prefer not to answer

 Don’t know

(c) To women?

 Yes

 No

 Prefer not to answer

 Don’t know

(d) To men?

 Yes

 No

 Prefer not to answer

 Don’t know

1. If you were going to be vaccinated, who would make this decision or give their permission?

- Your husband:  Yes  No  Prefer not to answer

- You:  Yes  No  Prefer not to answer

- Your father:  Yes  No  Prefer not to answer

- Your mother:  Yes  No  Prefer not to answer

1. If you were going to vaccinate your child(ren), who would make this decision or give their permission?

- Your husband:  Yes  No  Prefer not to answer

- You:  Yes  No  Prefer not to answer

- Your father:  Yes  No  Prefer not to answer

- Your mother  Yes  No  Prefer not to answer

1. The vaccine against HPV (to prevent cervical cancer) is given by:

 An injection into the cervix

 An injection into the arm

 Prefer not to answer

 Don’t know

1. You can protect yourself against HPV by:

 Using condoms

 Being vaccinated

 Both

 Prefer not to answer

 Don’t know

1. The vaccine against HPV (to prevent cervical cancer) is already available in Europe and in the United States. Would you want it to be available in Mali, as well?

 Yes

 No

 Prefer not to answer

 Don’t know

1. Would you want to participate in a vaccine trial to get the vaccine approved for use in Mali?

 Yes: For what reason?:

 No: For what reason?:

 Prefer not to answer

 Don’t know

1. Would you want your child(ren) to participate in a vaccine trial?

 Yes: For what reason?:

 No: For what reason?:

 Prefer not to answer

 Don’t know

1. If the vaccine is approved in Mali, would you want to get vaccinated?

 Yes

 No *(Directly go to Q 50)*

 Prefer not to answer

(b) Where would you want to be vaccinated? (Keep in mind that there are three shots, given at three separate appointments.)

 At the hospital

 At the CSCOM

 At school

 At home

 Other:

1. How would you like to be contacted for these three appointments?

 By phone call

 By text message

 By a visit to my house

 Other

 Prefer not to answer

 Don’t know

1. I would get vaccinated/I would vaccinate my child(ren) against cervical cancer:

 If the vaccine were free.

 If the vaccine were less expensive than  *____*.

 I would not get vaccinated or vaccinate my children against cervical cancer.
