# Supplementary material for: Knowledge, attitudes, practices and willingness to vaccinate in preparation for the introduction of HPV vaccines in Bamako, Mali
Source: PLoS One. 2017 Feb 13;12(2):e0171631. doi: 10.1371/journal.pone.0171631 (PMC5305061; doi:10.1371/journal.pone.0171631)
Supplement: S2 File — Questions asked after the education session are listed in S2 file. Every participant was asked questions 1–10 and 18–30. Only female participants were asked questions 11–17. Questions highlighted in green were also asked during the first interview before the education session. (DOC) [file pone.0171631.s002.doc]

##
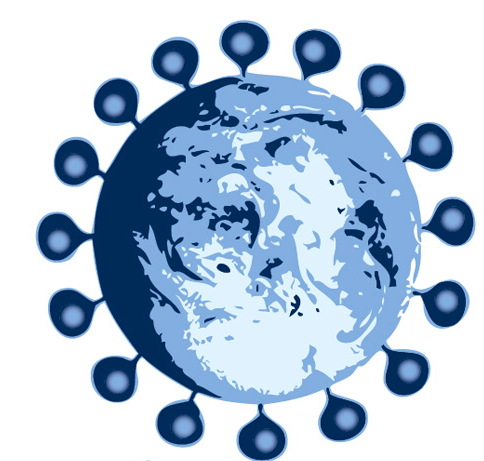
GAIA

**Global Alliance to Immunize against AIDS**

**146 Clifford Street (401) 453-2068**

**Providence, RI 02903 USA** [**www.GAIAvaccine.org**](http://www.GAIAvaccine.org/)

**Knowledge, Attitudes and Practices Study related to HPV and cervical cancer and Willingness to Participate in an HPV vaccine trial in the region of Bamako, Mali, West Africa**

Date :

ID Code :

**SECOND QUESTIONNAIRE**

**Part I : STIs and HPV**

1. Do you know what an STI is?

 Yes

 No *GO TO Q4*

 Prefer not to answer

2. Do you know how to protect yourself against STIs?

 Yes (Explain : ______________________________________)

 No

 Prefer not to answer

3. Do you know where to go to get tested for STIs?

 Yes (Where :__________________________________________)

 No

 Prefer not to answer

4. Do you know what HPV is?

 Yes (Please elaborate: ____________________________)

 No *GO TO PART 2*

 Prefer not to answer

5. Do you know one or more of the symptoms of HPV?

 Yes (Which ones : ____________________________________)

 No

 Prefer not to answer

6. Do you know how to protect yourself against HPV?

 Yes (Explain : ______________________________________)

 No

 Prefer not to answer

7. Please chose among the following answers:

 HPV affects women?

 HPV affects men?

 HPV affects adolescent girls ?

 HPV affects adolescent boys?

 Prefer not to answer

 Don’t know

8. Is HPV related to cervical cancer?

 Yes

 No

 Prefer not to answer

 I don’t know

9. Is HPV one of the main causes of cervical cancer?

 Yes

 No

 Prefer not to answer

 I don’t know

10a. Can cervical cancer be avoided if one is vaccinated against HPV?

 Yes

 No

 Prefer not to answer

 I don’t know

10b. Among women, is HPV one of the main causes of cervical cancer?

 Yes

 No

 Prefer not to answer

 I don’t know

*FOR MEN AND ADOLESCENT BOYS: GO TO PART 3*

**PART 2 : KNOWLEDGE ABOUT CERVICAL CANCER**

*(FOR WOMEN AND ADOLESCENT GIRLS ONLY)*

1. **Have you heard of cervical cancer?**

 Yes

 No

 Prefer not to answer

 I don’t know

12. **Do you know one or more of the symptoms or signs of cervical cancer?**

 Yes (Which ones : ____________________________________)

 No

 Prefer not to answer

 I don’t know

13. **Have you heard that there is free testing for cervical cancer in Mali?**

 Yes

 No

 Prefer not to answer

 I don’t know

14. **Do you know where to go to get tested (i.e. get a cervical exam)?**

 Yes (Where : _____________________)

 No

 Prefer not to answer

15. **True/False: A screening test tells you if you have cervical cancer?**

 True

 False

 Prefer not to answer

 I don’t know

16. **True or False: Cervical cancer is a cause of death among women?**

 True

 False

 Prefer not to answer

 I don’t know

17. **Do you know one or more of the causes of cervical cancer?**

 Yes (Which ones: _____________________________)

 No

 Prefer not to answer

 I don’t know

**PART 3 : KNOWLEDGE ABOUT VACCINES AND VACCINE TRIALS**

18. If the HPV vaccine were available in Mali should it be given to young adolescent girls before their first sexual relationships?

 Yes

 No

 Prefer not to answer

 I don’t know

19. If the HPV vaccine were available in Mali should it be given to young adolescent boys before their first sexual relationships?

 Yes

 No

 Prefer not to answer

 I don’t know

20. If the HPV vaccine were available in Mali should it be given to women?

 Yes

 No

 Prefer not to answer

 I don’t know

21. If the HPV vaccine were available in Mali should it be given to men?

 Yes

 No

 Prefer not to answer

 I don’t know

22. If you were going to be vaccinated, who would make this decision or give their permission?

Your husband:  Yes  No  Prefer not to answer

You:  Yes  No  Prefer not to answer

Your father:  Yes  No  Prefer not to answer

Your mother:  Yes  No  Prefer not to answer

23. If you were going to vaccinate your child(ren), who would make this decision or give their permission?

Your husband:  Yes  No  Prefer not to answer

You:  Yes  No  Prefer not to answer

Your father:  Yes  No  Prefer not to answer

Your mother  Yes  No  Prefer not to answer

24. What is the correct answer? The vaccine against HPV (to prevent cervical cancer) is given by an injection into the cervix or into the arm?

 An injection into the cervix

 An injection into the arm

 Prefer not to answer

 Don’t know

25. What is the correct answer? You can protect yourself against HPV by:

 Using condoms

 Being vaccinated

 Both

 Prefer not to answer

 Don’t know

26. The vaccine against HPV (to prevent cervical cancer) is already available in Europe and in the United States. Would you want it to be available in Mali, as well?

 Yes

 No

 Prefer not to answer

 Don’t know

27. Would you want to participate in a vaccine trial to get the vaccine approved for use in Mali?

 Yes: For what reason?:

 No: For what reason?:

 Prefer not to answer

 Don’t know

28. Would you want your child(ren) to participate in a vaccine trial?

 Yes: For what reason?:

 No: For what reason?:

 Prefer not to answer

 Don’t know

29. If the vaccine is approved in Mali, would you want to get vaccinated?

*FOR ADULTS: Would you want your child/children to get vaccinated?*

 Yes

 No

 Prefer not to answer

 I don’t know

30. I would get vaccinated/I would vaccinate my child(ren) against cervical cancer:

 If the vaccine were free.

 If the vaccine were less expensive than  *____*.

 I would not get vaccinated or vaccinate my children against cervical cancer.

 Prefer not to answer

 I don’t know
