# Supplementary material for: Knowledge, attitudes, practices and willingness to vaccinate in preparation for the introduction of HPV vaccines in Bamako, Mali
Source: PLoS One. 2017 Feb 13;12(2):e0171631. doi: 10.1371/journal.pone.0171631 (PMC5305061; doi:10.1371/journal.pone.0171631)
Supplement: S3 File — Age-adapted informed consent forms were used in this study. Page 1–2 of S3 file shows the consent form used for adults > 18 years old. Pages 3–6 shows consent forms proposed to adolescents < 18 years old and their legal guardian. (DOC) [file pone.0171631.s003.doc]

**Adulte**

**Titre de l’étude:** **Etude des Connaissances, Comportements Attitudes et Pratiques liés au cancer du col de l’utérus et perspective d’essai vaccinal contre le HPV dans la région de Bamako - Mali Afrique de L’Ouest**

**Nom de l’institution**: Fondation Gaia, Hippodrome, Rue 224 Porte 1803 Bamako Mali

**Investigateur Principal:** Dr Karamoko Tounkara

Tel: 76495886

Email: tounkara.karamoko@gmail.com

**Quelles sont les implications d’une participation à l’étude?**

Votre participation à cette étude est volontaire et anonyme. Personne n’aura accès à ses informations en dehors des investigateurs principaux. Vous n’êtes pas obligé(e) de participer à cette étude.

En signant ce consentement vous nous donnez la permission d’utiliser vos réponses pour la collecte, l’analyse et l’exploitation de nos données permettant la rédaction et la publication d’articles médicaux.

Risques et bénéfices liés à votre participation: Les risques liés à votre participation à ce questionnaire sont mineurs. Vous n’êtes pas obligé(e) de répondre à toutes les questions.

**Les bénéfices liés à votre participation sont les suivants:**

Vos réponses et votre opinion vont nous permettre d’évaluer les Connaissances, Attitudes et Pratiques au sujet du Cancer du col de l'utérus et la volonté de participer à un essai vaccinal contre le HPV à Mekin-Sikoro.

Pour toutes questions ou réclamations merci de contacter l’investigateur principal Dr Tounkara Karamoko

(Tel : 76495886). Vous pouvez aussi contacter le président du Comité d’Ethique de la FMPOS au Point G, Bamako (Tel : 20225277).

**Déclaration de consentement**

Je, soussigné(e) Mr/Mme____________________________________, résident de_______________, confirme que les objectifs et méthodes de l’étude Connaissances, Attitudes et Pratiques sur le Cancer du col de l'uterus et perspective d'un essai vaccinal contre le HPV à Mekin-sikoro m’ont été expliqués et que la possibilité de poser des questions sur l’étude m’a été donnée.

J’ai eu l’assurance que l’investigateur garantira la confidentialité de toute information sensible recueillie. J’ai été informé(e) des risques et des avantages liés à ma participation. J’ai pris connaissance des contraintes de temps que ma participation pourrait me poser. J’ai été informé(e) que ma participation n’implique pas de paiement ni à moi ni à un membre de ma famille. Je comprends que la participation est volontaire et que j’ai le droit de me retirer de l’étude à tout moment si je le souhaite.

J’accepte volontairement de prendre part à cette étude : /___/ Oui /___/ Non.

**Signature /empreinte digitale :** __________________________________ **Date**:_____________

**Nom et Prénom de l’investigateur**:________________________________

Anne De Groot, MD Dr Tounkara Karamoko

Fondatrice et Directrice Scientifique Directeur Executif

**FICHE de CONSENTEMENT**

**Tuteur**

**Titre de l’étude:** **Etude des Connaissances, Comportements Attitudes et Pratiques liés au cancer du col de l’utérus et perspective d’essai vaccinal contre le HPV dans la région de Bamako - Mali Afrique de L’Ouest**

**Nom de l’institution**: Fondation Gaia, Hippodrome, Rue 224 Porte 1803 Bamako Mali

**Investigateur Principal:** Dr Karamoko Tounkara

Tel: 76495886

Email: tounkara.karamoko@gmail.com

**Quelles sont les implications d’une participation à l’étude?**

La participation à cette évaluation est volontaire et anonyme**.** Personne n’aura accès à ses informations en dehors des investigateurs principaux. L’adolescent que vous accompagnez n’est pas obligé de participer à cette étude.

En signant ce consentement vous nous donnez la permission d’utiliser les réponses pour la collecte, l’analyse et l’exploitation de nos données permettant la rédaction et la publication d’articles médicaux.

Risques et bénéfices liés à la participation à cette étude: Les risques liés à la participation à ce questionnaire sont mineurs. L’adolescent n’est pas obligé de répondre à toutes les questions.

**Les bénéfices liés à l**a **participation sont les suivants:** Les réponses et les opinions exprimées vont nous permettre d’évaluer les Connaissances, Attitudes et Pratiques au sujet du Cancer du col de l'utérus et la volonté de participer à un essai vaccinal contre le HPV à Mekin-Sikoro.

Pour toutes questions ou réclamations merci de contacter l’investigateur principal Dr Tounkara Karamoko (Tel : 76495886). Vous pouvez aussi contacter le président du Comité d’Ethique de la FMPOS au Point G, Bamako (Tel : 20225277).

**Déclaration de consentement**

Je, soussigné(e) Mr/Mme____________________________________, tuteur de _________________ ,

résident de_______________, confirme que les objectifs et méthodes de l’étude Connaissances, Attitudes et Pratiques sur le Cancer du col de l'uterus et perspective d'un essai vaccinal contre le HPV à Mekin-sikoro m’ont été expliqués et que la possibilité de poser des questions sur l’étude m’a été donnée.

J’ai eu l’assurance que l’investigateur garantira la confidentialité de toute information sensible recueillie. J’ai été informé(e) des risques et des avantages liés à la participation de l’adolescent que j’accompagne. J’ai pris connaissance des contraintes de temps que sa participation pourrait lui poser. J’ai été informé(e) que sa participation n’implique pas de paiement ni à lui ni à moi. Je comprends que sa participation est volontaire et qu’il a le droit de se retirer de l’étude à tout moment s’il le souhaite.

J’accepte volontairement que l’adolescent(e) prenne part à cette étude : /___/ Oui /___/ Non.

**Signature /empreinte digitale :** __________________________________ **Date**:_____________

**Nom et Prénom de l’investigateur**:________________________________

Anne De Groot, MD Dr Tounkara Karamoko

Fondatrice et Directrice Scientifique Directeur Executif

**FICHE de CONSENTEMENT**

**Adolescent**

**Titre de l’étude:** **Etude des Connaissances, Comportements Attitudes et Pratiques liés au cancer du col de l’utérus et perspective d’essai vaccinal contre le HPV dans la région de Bamako - Mali Afrique de L’Ouest**

**Nom de l’institution**: Fondation Gaia, Hippodrome, Rue 224 Porte 1803 Bamako Mali

**Investigateur Principal:** Dr Karamoko Tounkara

Tel: 76495886

Email: tounkara.karamoko@gmail.com

**Quelles sont les implications d’une participation à l’étude?**

Votre participation à cette évaluation est volontaire et anonyme**.** Personne n’aura accès à ses informations en dehors des investigateurs principaux. Vous n’êtes pas obligé(e) de participer à cette étude.

En signant ce consentement vous nous donnez la permission d’utiliser vos réponses pour la collecte, l’analyse et l’exploitation de nos données permettant la rédaction et la publication d’articles médicaux.

Risques et bénéfices liés à votre participation: Les risques liés à votre participation à ce questionnaire sont mineurs. Vous n’êtes pas obligé(e) de répondre à toutes les questions.

**Les bénéfices liés à votre participation sont les suivants:**

Vos réponses et votre opinion vont nous permettre d’évaluer les Connaissances, Attitudes et Pratiques au sujet du Cancer du col de l'utérus et la volonté de participer à un essai vaccinal contre le HPV à Mekin-Sikoro.

Pour toutes questions ou réclamations merci de contacter l’investigateur principal Dr Tounkara Karamoko (Tel : 76495886). Vous pouvez aussi contacter le président du Comité d’Ethique de la FMPOS au Point G, Bamako (Tel : 20225277).

**Déclaration de consentement**

Je, soussigné(e) ____________________________________, résident de_______________, confirme être accompagné(e) par un tuteur qui a donné son accord pour ma participation à cette étude. Je confirme également que les objectifs et méthodes de l’étude Connaissances, Attitudes et Pratiques sur le Cancer du col de l'uterus et perspective d'un essai vaccinal contre le HPV à Mekin-sikoro m’ont été expliqués et que la possibilité de poser des questions sur l’étude m’a été donnée.

J’ai eu l’assurance que l’investigateur garantira la confidentialité de toute information sensible recueillie. J’ai été informé(e) des risques et des avantages liés à ma participation. J’ai pris connaissance des contraintes de temps que ma participation pourrait me poser. J’ai été informé que ma participation n’implique pas de paiement ni à moi ni à un membre de ma famille. Je comprends que la participation est volontaire et que j’ai le droit de me retirer de l’étude à tout moment si je le souhaite.

J’accepte volontairement de prendre part à cette étude : /___/ Oui /___/ Non.

**Signature /empreinte digitale :** __________________________________ **Date**:_____________

**Nom et Prénom de l’investigateur**:________________________________

Anne De Groot, MD Dr Tounkara Karamoko

Fondatrice et Directrice Scientifique Directeur Executif
