# Supplementary material for: Knowledge, attitudes, practices and willingness to vaccinate in preparation for the introduction of HPV vaccines in Bamako, Mali
Source: PLoS One. 2017 Feb 13;12(2):e0171631. doi: 10.1371/journal.pone.0171631 (PMC5305061; doi:10.1371/journal.pone.0171631)
Supplement: S4 File — A three- days training was provided for interviewers before initiation of the study. The training manual is displayed in S4 file, in French. (DOCX) [file pone.0171631.s004.docx]

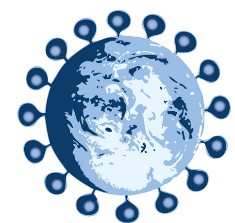


## GAIA

**Global Alliance to Immunize against AIDS**

**146 Clifford Street, Prrovidence, RI 02903 USA**

**(401) 453-2068** [**www.GAIAvaccine.org**](http://www.GAIAvaccine.org)

**Connaissances, Attitudes et Pratiques d’étude lies au HPV à cancer du col et la volonté de participer à un essai de vaccin contre le HPV dans la région de Bamako, Mali Afrique de l’Ouest**

**LA FORMATION DES INTERVIEWEURS**

**JOUR 1**

Bienvenue a l’étude HPV CAP et WTP

Objet de l’étude HPV CAP et WTP

Vue d’ensemble du HPV et cancer du col

Design de l’étude HPV CAP et WTP

Rôle of l’intervieweur

**JOUR 2**

Intervieweur Protocole

La confidentialité des participants

Consentement Protocole

**JOUR 3**

Pratique d’entrevue avec les questionnaires

**Bienvenue a l’étude Human Papillomavirus Connaissances, Attitudes, et Pratiques et volonté de participer à un essai de vaccin contre le HPV**

Cher Monsieur/Madame,

Bienvenue a l’étude Human Papillomavirus (HPV) connaissances, attitudes, et pratiques et volonté de participer (HPV CAP et VDP), et merci pour votre aide. Votre rôle d’intervieweur aidera notre équipe a notre objectif de porter le vaccine contre le HPV au Mali. Tous les enquêteurs sur notre équipe complète de formation avant de commencer l’étude.

La formation suivra ce manuel. Nous allons d’abord discuter l’étude et de HPV et cancer du col. Apres ça, nous allons discuter du protocole pour être un intervieweur dans cette étude. Il s’agir notamment de la façon d’obtenir le consentement des deux adultes et adolescents, et comment entretenir la vie privée des participants. Enfin, nous allons nous exercer s’interroger mutuellement, puis ont on défi interview.

Il est très important que vous deveniez experts en la matière, et que vous etes a l’aise a l’administration du questionnaire. À tout moment, si vous avez une question, s’il vous plait demander a l’entraîneur. Si vous préférez demander an prive, vous pouvez demander a l’entraîneur après la session de formation.

Parce qu’il est important pour vous de devenir un expert en la matière, nous aurons a court examens pour s’assurer que notre formation est complète. Nous allons poursuivre la formation que tout le monde passé les examens a court.

Nous espérons que vous trouverez la formation intéressante. Bienvenue a l’équipe de GAIA!

**Objet de l’étude HPV CAP et WTP**

Il s’agit d’une connaissance, l’attitude, et les pratiques (CAP), et la volonté de participer (VDP) a l’étude. Cette étude a trois objectifs :

1. Nous aimerions déterminer ce que les gens savent déjà sur le HPV, et le fait que le HPV peut causer le cancer du col chez les femmes.
2. Nous aimerions apprendre aux gens a propos du HPV, et le fait que le HPV peut causer le cancer du col chez femmes.
3. Nous aimerions apprendre aux gens qu’il existe un vaccin contre le HPV, et qu’il est utilisé partout dans le monde. Nous aimerions savoir si les gens au Mali souhaite recevoir cette vaccination aussi.

Pour réaliser ce projet, nous procéderons à des entrevues et des séances de formation avec des personnes dans le communauté. Le HPV est une infection très commune sexuellement transmissible (ST). Nous avons besoin d’apprendre sur le HPV, et comment parler de ce sujet sensible quand on donne des interviews.

Que les intervieweurs, notre travail consiste à :

1. Nous devons devenir des experts sur le HPV, et comment il est transmis et empêche. Nous avons aussi besoin de comprendre la maladie qui est parfois causées par le HPV.
2. Nous devons être à l’aise de parler sur le HPV avec les gens dans la communauté.
3. Nous devons apprendre à protéger les personnes qui participent a notre étude. Cela signifie que nous devons apprendre à obtenir le consentement éclaire, et la façon de conserver toutes les informations que nous apprenons au cours des entretiens confidentiels.

**Vue d’ensemble du HPV et cancer du col**

1. **Virus du papillome humain (HPV)**


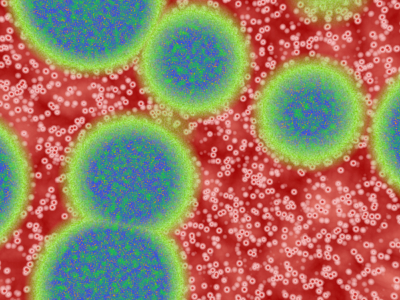
Papillomavirus génitaux de l'homme (également appelé HPV) est l'infection la plus commune sexuellement transmissibles (IST). Il y ‘a plus de 40 types de HPV qui peuvent infecter les parties génitales des hommes et des femmes. Ces types de HPV peuvent aussi infecter la bouche et la gorge. La plupart des personnes infectées par le HPV ne savent même pas qu'elles en sont parce que parfois il n'y’a pas de symptômes.

Le HPV n'est pas la même que l'herpès ou le VIH (le virus qui cause le SIDA). Ce sont tous les virus qui peuvent être transmis lors de rapports sexuels, mais ils parlent des symptômes différents et des problèmes de santé.

*La Figure 1. Le virus HPV agrandi.*

1. **Infection par le HPV**

La plupart des gens par le HPV ne développent pas de symptômes ou de problèmes de santé de lui. Dans 90% des cas, le système immunitaire de l'organisme autorise le VPH naturellement dans les deux ans.

Mais parfois, certains types de HPV peuvent causer des verrues génitales chez les mâles et les femelles. Rarement, ces types peuvent également causer des verrues dans la gorge - une condition appelée papillomatose respiratoire récurrente ou PPC.

D'autres types de VPH peuvent causer le cancer du col. Ces types peuvent également provoquer des cancers d'autres, moins courants mais graves, y compris les cancers de la vulve, du vagin, du pénis, l'anus, et la tête et du cou (la langue, les amygdales et la gorge).

Les types de HPV qui peuvent causer des verrues génitales ne sont pas les mêmes que les types qui peuvent causer le cancer. Il n'y a aucun moyen de savoir quelles personnes infectées par le HPV ira se développer un cancer ou autres problèmes de santé.

**Les signes et symptômes des problèmes liés au HPV:**


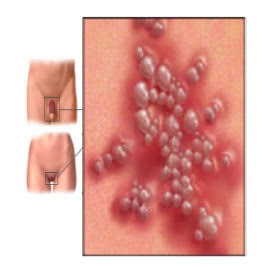
Les verrues génitales apparaissent généralement comme une petite bosse ou des groupes de bosses dans la région génitale. Petites ou grandes, surélevées ou plates, ou en forme de chou-fleur. Fournisseurs de soins de santé peut diagnostiquer les verrues en regardant les parties génitales au cours d'une visite au bureau. Les verrues peuvent apparaître dans les semaines ou des mois après un contact sexuel avec un partenaire infecté, même si le partenaire infecté n'a pas de signes de verrues génitales. Si elle n'est pas traitée, les verrues génitales peuvent disparaître, restent inchangées, ou augmentation de la taille ou le nombre. Ils ne vont pas se transformer en cancer.

*La Figure 2. Les verrues génitales*

HPV peuvent causer des cellules normales sur la peau infectée à son tour anormale. La plupart du temps, vous ne pouvez pas voir ou de sentir ces changements cellulaires. Dans la plupart des cas, l'organisme lutte contre le VPH naturellement et les cellules infectées, puis revenir à la normale. Mais dans les cas où l'organisme ne lutte pas contre le HPV, le HPV peut causer des changements visibles dans la forme de verrues génitales ou le cancer. Les verrues peuvent apparaître dans les semaines ou des mois après l'obtention du HPV. Cancer prend souvent des années à se développer après le HPV.

**Cancer du col:**

Cancer du col n'a habituellement pas de symptômes jusqu'à ce qu'il soit assez avancé. Pour cette raison, il est important pour les femmes d'obtenir un dépistage régulier du cancer du col. Les tests de dépistage, on trouve les premiers signes de la maladie afin que les problèmes peuvent être traités rapidement, avant qu'ils ne jamais se transformer en cancer.


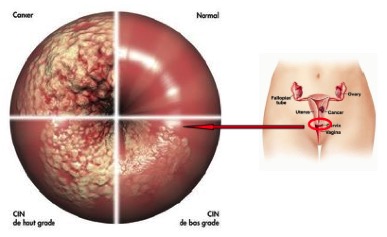
23,2 pour 100000 femmes ont le cancer du col au Mali. Toutes les 1491 années plus de femmes auront cancer du col a cause de l’infection au HPV.

Autres cancers liés au HPV peut-être pas des signes où des symptômes jusqu'à ce qu'ils soient avancés et difficiles à traiter. Il s'agit notamment de cancers de la vulve, du vagin, du pénis, l'anus, et la tête et du cou.

*La Figure 3. Cancer du col*

1. **Transmission du HPV**

Le HPV est transmis par contact génital, le plus souvent lors de rapports sexuels vaginaux et anaux. Le HPV peut également être transmis lors de rapports sexuels oraux et contact génital-génital. Le HPV peut être transmis entre les droites et les partenaires de même sexe, même lorsque le partenaire infecté n'a pas de signes ou de symptômes.

Une personne peut avoir contre le HPV, même si ans se sont écoulés depuis qu'il a eu un contact sexuel avec une personne infectée. La plupart des personnes infectées ne savent pas qu'ils sont infectés ou qu'ils transmettent le virus à un partenaire sexuel. Il est également possible d'obtenir plus d'un type de HPV.

Très rarement, une femme enceinte avec HPV génitaux peuvent transmettre le HPV à son bébé pendant l'accouchement.

1. **Prévention de l’infection HPV**

Les vaccins peuvent protéger les hommes et les femmes contre certains des types les plus courants de VPH. Ces vaccins sont administrés en trois injections. Il est important d'obtenir les trois doses pour obtenir la meilleure protection. Les vaccins sont plus efficaces lorsqu'ils sont administrés avant la première d'une personne contact sexuel, quand il ou elle pourrait être exposées au HPV.

**Design de l’étude HPV CAP et VDP**

Premièrement, nous allons devenir des experts dans le sujet du HPV et le cancer du col. Alors nous commençons à bien mener l'étude pilote, qui comprend la réalisation de 50 entrevues. Nous irons comme une équipe de cinq personnes pour mener les entrevues dans les maisons des membres de la communauté. Dans notre équipe, nous aurons deux femmes et deux hommes d'âges différents. Les femmes plus âgées seront interviewer les femmes et les jeunes femmes vont interroger les adolescentes. Les hommes plus âgés intervieweront les hommes, les jeunes gens vont interroger l'adolescent.

**Rôle of l’intervieweur**

Que les enquêteurs, il est de notre devoir d'expliquer l'étude aux participants.

Pourquoi voulons-nous de poser ces question?

- Nous aimerions savoir si les gens ont entendu parler du HPV, et ce qu'ils savent à ce sujet.
- Nous aimerions savoir si les gens ont entendu parler du cancer du col, et ce qu'ils savent à ce sujet.
- Nous aimerions savoir si les gens sont à risque d'infection par le HPV.
- Nous aimerions savoir si les gens aimeraient recevoir un vaccin pour les protéger contre le HPV.
- Nous aimerions savoir, si ce n'est pas un individu, qui prendrait la décision pour eux de se faire vacciner.
- Nous aimerions savoir où les gens aimeraient recevoir le vaccin.

Une fois que nous expliquer l'étude à une personne, nous leur demanderons la permission de les interroger. Expliquant l'étude ainsi aux participants augmente leur intérêt, et peut augmenter leur volonté de remplir le questionnaire.

**Si la personne est un adolescent(es)**, nous demandons que l'adolescent(es) **et** le parent donner leur consentement pour l'adolescent de participer à l'entrevue.

**Si la personne est un adulte**, vous demandez-leur de donner leur consentement de participer à l'entrevue.

De donner son consentement, la personne doit comprendre l'étude, de comprendre que leurs réponses sont confidentielles, et qu'ils sont libres d'arrêter, même après avoir accepté de participer. Il est de notre devoir de s'assurer que la personne comprend toutes ces choses.

**JOUR 2**

**Intervieweur Protocole**

Si vous obtenir le consentement, vous pouvez procéder à l'entrevue. s'il vous plaît lire les questions exactement comme elles sont écrites sur le questionnaire. Lorsque les réponses des répondants, enregistrer leur réponse avant de passer à la question suivante.

Après avoir terminé, le premier questionnaire, s'il vous plaît passer dix minutes va sur le HPV et le cancer du col forme d'enseignement avec le participant. Ensuite, terminer l'entretien en leur donnant le dernier questionnaire. Nous espérons que votre capacité à éduquer les participants amélioreront leur connaissance du HPV et le cancer du col.

Si le participant vous demande de l'aide lors de l'entretien, reportez-vous au participant de Danielle et elle vous aidera à faire une référence appropriée.

Si un participant ne comprenez pas une question, vous pouvez d'abord répéter la question. Si la question n'est toujours pas clair, vous pouvez expliquer la signification de celle-ci avec des mots différents. Si cela se produit, s'il vous plaît prendre note de ce.

Le participant peut sauter toutes les questions qu'ils veulent. Aussi, ils peuvent quitter l'entrevue à tout moment qu'ils veulent. Un adulte qui a donné leur consentement pour un adolescent peut aussi arrêter l'interview d'un adolescent à tout moment.

**La confidentialité des participants**

La confidentialité des participants sont protégés dans toute la mesure de la loi. Aucun nom ou autre identification personnelle sera écrit sur le questionnaire. Au contraire, nous allons utiliser un code numérique pour identifier l'ensemble de nos données. Protéger la confidentialité des participants signifie également que vous ne doit jamais discuter de tout entretien de manière que la personne interrogée n'a pu être identifié.
